# A cervical cancer biorepository for pharmacogenomics research in Zimbabwe

**DOI:** 10.1186/s12885-022-10413-w

**Published:** 2022-12-16

**Authors:** Oppah Kuguyo, Nyasha Chambwe, Charles F. B. Nhachi, Nomsa Tsikai, Collet Dandara, Alice Matimba

**Affiliations:** 1 Clinical Pharmacology Department, University of Zimbabwe College of Health Sciences, Avondale, Mazowe Street, Harare, Zimbabwe; 2 Institute of Molecular Medicine, Feinstein Institutes for Medical Research, Northwell Health, Manhasset, NY USA; 3 Department of Oncology, University of Zimbabwe College of Health Sciences, Harare, Zimbabwe; 4 Pharmacogenomics and Drug Metabolism Research Group, Division of Human Genetics, Department of Pathology & Institute of Infectious Diseases and Molecular Medicine, Faculty of Health Sciences, University of Cape Town, Cape Town, South Africa

**Keywords:** Cervical cancer, Cancer Biobanks, Biorepository, Africa, Zimbabwe, Pharmacogenomics, Genomics

## Abstract

**Background:**

Research infrastructures such as biorepositories are essential to facilitate genomics and its growing applications in health research and translational medicine in Africa. Using a cervical cancer cohort, this study describes the establishment of a biorepository consisting of biospecimens and matched phenotype data for use in genomic association analysis and pharmacogenomics research.

**Method:**

Women aged > 18 years with a recent histologically confirmed cervical cancer diagnosis were recruited. A workflow pipeline was developed to collect, store, and analyse biospecimens comprising donor recruitment and informed consent, followed by data and biospecimen collection, nucleic acid extraction, storage of genomic DNA, genetic characterization, data integration, data analysis and data interpretation. The biospecimen and data storage infrastructure included shared -20 °C to -80 °C freezers, lockable cupboards, secured access-controlled laptop, password protected online data storage on OneDrive software. The biospecimen or data storage, transfer and sharing were compliant with the local and international biospecimen and data protection laws and policies, to ensure donor privacy, trust, and benefits for the wider community.

**Results:**

This initial establishment of the biorepository recruited 410 women with cervical cancer. The mean (± SD) age of the donors was 52 (± 12) years, comprising stage I (15%), stage II (44%), stage III (47%) and stage IV (6%) disease. The biorepository includes whole blood and corresponding genomic DNA from 311 (75.9%) donors, and tumour biospecimens and corresponding tumour DNA from 258 (62.9%) donors. Datasets included information on sociodemographic characteristics, lifestyle, family history, clinical information, and HPV genotype. Treatment response was followed up for 12 months, namely, treatment-induced toxicities, survival vs. mortality, and disease status, that is disease-free survival, progression or relapse, 12 months after therapy commencement.

**Conclusion:**

The current work highlights a framework for developing a cancer genomics cohort-based biorepository on a limited budget. Such a resource plays a central role in advancing genomics research towards the implementation of personalised management of cancer.

**Supplementary Information:**

The online version contains supplementary material available at 10.1186/s12885-022-10413-w.

## Introduction

Biorepositories have advanced genomics research in the developing world, by providing access to a readily available pool of systematic, harmonised, and high-quality biospecimens and associated phenotype data [1, 2]. In clinical research, adequate recruitment is critical to ensure sufficient study power and to competently answer the research question [2]. Equally so, insufficient participant retention during recruitment affects the conclusiveness of findings [3]. Studies in resource-limited settings report recruitment challenges for clinical research due to conservative cultural mindsets, complex study protocols that participants cannot understand during informed consent, incomplete clinical information captured by physicians, poor health-seeking behaviour which results in patients not attending clinics after diagnosis, socioeconomic factors, and inadequate resources for prolonged recruitment [3–13]. Thus, readily available data and biospecimens in well-protected biorepositories are essential to address context-specific complexities and expedite research for understanding genomic mechanisms in cancer. This will aid in reducing research costs for subsequent studies, especially where resources are limited. In addition, the relatively modest cost, prolonged time, complex ethical compliance requirements, and expertise needed for comprehensive biospecimen, and data collection makes a case for coordinated efforts to develop biorepositories that apply standardised data collection procedures for disease-specific cohorts.

Recognising the unique diversity of the African genome in comparison to other populations [14], and the potential to identify novel associations with disease, there is a growing demand for resources to facilitate research to determine the role of genomics research across Africa. Therefore, biorepositories are becoming critical resources to determine the role of genomics in disease susceptibility and treatment response using African populations. This study focused on establishing a biorepository for female reproductive cancers, starting with cervical cancer since it is a major health concern in Zimbabwe due to constraints in the public health system and challenges faced in implementing prevention and screening strategies [15]. Consequently, most women are diagnosed late, associated with poor prognosis and high mortality [15]. Therefore, focused interventions to upscale prevention and early detection are needed in addition to expanded clinical research for better treatment and prognosis.

Several cancer biorepositories exist in the form of consortia and large-scale clinical studies for biomarker research [16, 17]. The International Agency for Research on Cancer (IARC) has established a global cancer biorepository with 6 million biospecimens from 600 000 donors, towards applying innovative methodologies to understand causality and design early detection and preventive measures against cancer [18]. In addition, various projects under the Human Hereditary and Health in Africa (H3Africa) consortium are studying cancer in African populations while also establishing cancer biorepositories using harmonised guidelines [19]. However, these biorepositories often focus on addressing issues primarily related to the specific environment and research questions or have limited access to biospecimens and data.

In Zimbabwe, the few available biorepositories are dominated by biospecimens and data on infectious diseases such as malaria, tuberculosis, and HIV [20–22]. The largest study for cervical cancer cohorts in Zimbabwe has mostly focused on the relationship with HIV and other reproductive infections [23]. Furthermore, there are also studies that have attempted to elucidate the association of genetic polymorphisms with cancer susceptibility in Zimbabwe [24–28], but these datasets may not have been established using harmonised methods, which can limit genomic applications.

Since many African countries are only starting to establish basic capacity and building resources to support research in genomics of various diseases, scientists are also developing relevant frameworks and building capacity [29–31]. Biorepositories could play a central role in building local and regional capacity, and fostering collaborative research, particularly in cancer pharmacogenomics where the benefits have been well demonstrated in predicting response to chemotherapies.

With over 40 genetic polymorphisms approved as biomarkers for various pharmacological drugs, studies in African populations are lagging. To address this limitation, a biobank and pharmacogenomics database of African populations was established, however mainly focused on the determination of novel variants and genotype frequencies, thereby lacking linkage with clinical phenotypes [32–34]. To facilitate the pharmacogenomics of cervical cancer research and potentially contribute to the development of targeted therapies, this work builds upon existing efforts to develop resources and capacity for cancer pharmacogenomics research, biomarker discovery and personalised medicine using a cervical cancer cohort in Zimbabwe. Data collected included risk factors, treatment administered and high-quality genomic DNA that can be used for future characterisation of cervical cancer susceptibility and treatment response biomarkers. The objectives of this paper are to describe the steps taken in establishing a biorepository for cancer with limited resources, outcomes, and future potential utility of the resources in capacity development of pharmacogenomics research of female reproductive cancers.

## Method

This biorepository was established as a key objective for a research project that aimed to conduct pharmacogenomics studies for cervical cancer among Zimbabwean women.

### Donor recruitment

Donors were recruited prospectively between June 2016 and January 2019, at a single site, the Parirenyatwa Group of Hospitals Radiotherapy and Chemotherapy Centre (RTC). Donors in the biorepository were followed up over a 12-month period. Potential donors were identified through the RTC registry and checked for eligibility to donate. To be considered eligible, individuals had to have histologically confirmed cervical cancer, be aged > 18 years, be newly diagnosed with no previous history of anti-cancer treatments and be scheduled to receive radical anti-cancer interventions. Women scheduled to receive palliative care were excluded from the biorepository.

### Informed consent

Broad consent was administered, stipulating that the donor agreed for their biospecimens and associated data to be stored and used for future genomics research. The consent form also stated that the biorepository was not going to be used for financial gain, but instead for biomedical advancement. In the consenting process, all individuals were informed of the purpose of the biorepository to establish the collectiveness of goals and mutual trust between the researchers and donors. Additionally, consent to conduct follow-up tests for treatment-induced toxicities (TITs) of interest at successive clinic visits was requested. Consent and enrolment were conducted while the donor was waiting to see the doctor. Consent forms were available in the three main languages spoken in Zimbabwe: Shona, Ndebele and English [35].

### Overall strategy of data collection

**Fig. 1:**
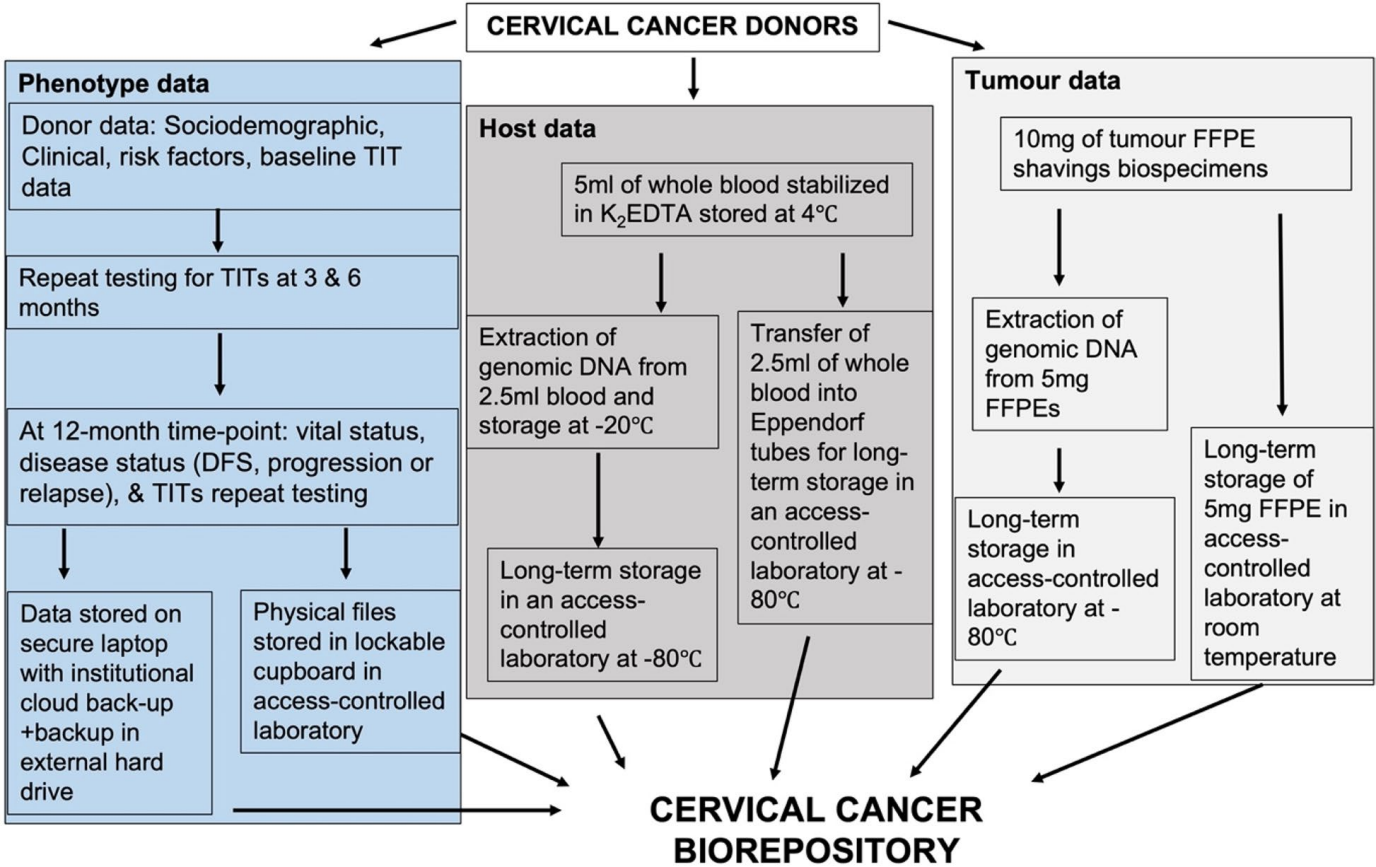
Schematic overview of the cervical cancer biorepository. Footnote: TITs- Treatment-induced toxicities, DFS- disease free survival, K2EDTA- potassium ethylene diamine tetraacetic acid, FFPE- formalin fixed paraffin embedded preservation

All donors were assigned a unique identifier to be used to associate phenotype and biospecimen data. The biorepository is made up of a phenotype and biospecimen data component. The phenotype component consists of sociodemographic, clinical, risk factor, TITS, vital status, and disease status – survival, disease progression or relapse data (Fig. 1). The biospecimen component encompasses whole blood (host data) and archived tumour biospecimens (tumour data). Storage of all components is in an access-regulated laboratory.

### Donor demographic and clinical data collection

Data were collected by a research assistant with Good Clinical Practice certification [36]. Prior to commencement, the research assistant was trained in administering informed consent, collecting data, and performing follow-up tests for TITs to ensure reliable data collection. A de-identified standardised paper-based data collection tool was used to record demographic and phenotype data. At recruitment, sociodemographic, behavioural, gynaecological, and family history variables were collected along with tumour histopathology and treatment regimens (Table 1).

**Table 1:** Data collected in the cervical cancer bioresource

Classification of factor	List of variables collected
Sociodemographic	Age, ancestral origin, marital status, residency, occupation
Lifestyle or behavioural	Alcohol consumption, tobacco smoking, physical exercise history, age at sexual debut, number of sexual partners
Gynaecological	Menopausal status, age of menopause, age of menarche, history of STI, HIV status, menstruation regularity, age at first contraceptive, type of contraceptive, duration on contraceptive, parity, age at first conception,
Family history	Number of siblings, number of children, history of cancer in family
Clinical	Weight, height, presenting symptoms, respiratory system status, central nervous system status, rectal system status, comorbidities, co-medications
Tumour histopathology	FIGO stage, tumour size, histological type, presence of metastatic disease, presence of hydronephrosis
Treatment administered	Type of treatment, drug, dose, number of cycles, treatment notes *e.g*. delays in therapy, subsequent clinic visit dates
Outcome of treatment at 12 months	Disease free survival, relapse, disease progression, renal function, presence of deep vein thrombosis, presence of vesicovaginal fistula
Other	History of herbal medicines, personal history of cancer, circumcised sexual partner

*STI* Sexually transmitted infection, *HIV* Human immunodeficiency virus, *FIGO* International federation of gynecology and obstetrics, *DVT* Deep vein thrombosis, *VVF* Vesicovaginal fistula

Donors were also followed up over 12 months for survival, disease progression, cervical cancer relapse, and TITs at 3, 6 and 12 months. TITs of interest included deep vein thrombosis (DVT), ototoxicity, peripheral neuropathy, radiation proctitis, renal injury, vaginal stenosis, vesicovaginal fistula (VVF) and vestibulopathy at specific time points (Table 2). Subsequent follow-up visits were scheduled to coincide with prescribed doctor’s visits. Consistent data formats, checking for completeness of data collected at the recruitment site, and cross-checking of data capturing, were done at the end of each day by the data capturer; and on a weekly basis by the data management team for quality assurance. The paper-based data collection sheets were filed in a folder and placed in a lockable cabinet for long-term storage. All phenotype data was also abstracted onto an electronic case report form on Microsoft Excel (Microsoft Corporation, Redmond, Washington, USA). Backup of this electronic phenotype data is stored on a hard drive that is securely kept in a lockable cupboard. A soft copy of the data is uploaded onto a commercially available cloud storage (OneDrive, Microsoft Corporation, Redmond, Washington, USA).

**Table 2:** Follow-up schedule for the donors in the cervical cancer bioresource

TIT	Follow-up period (months)			
	**Pre-treatment**	**3**	**6**	**12**
**DVT**^**a**^	✓	**✗**	**✗**	✓
**Renal injury**^**a**^	✓	✓	✓	✓
**Peripheral Neuropathy**	✓	✓	✓	✓
**Ototoxicity**	✓	✓	✓	✓
**Radiation Proctitis**^**a**^	✓	**✗**	**✗**	✓
**Vaginal stenosis**^**a**^	✓	**✗**	**✗**	✓
**VVF**^**a**^	✓	**✗**	**✗**	✓
**Vestibulopathy**	✓	✓	✓	✓

^a^Abstracted from patient clinical data; ✓**:** followed up; **✗:** not followed up

### Biospecimen component

#### Blood collection, processing, and storage

Five millilitres of peripheral blood were obtained before donors received any form of anti-cancer therapy. Blood was drawn using jugular venepuncture and the blood was immediately stabilised in potassium ethylenediamine tetra-acetic acid anticoagulant vacutainer tubes. Blood was mixed by inversion and immediately kept at 4 °C for a maximum of 4 h until transfer to the laboratory for long-term storage and processing. The blood was aliquoted into Eppendorf tubes, and half of the collected whole blood (2.5 ml) was stored fresh frozen at -20 °C for less than 1 month and moved to -80 °C as unfractionated blood components of the biorepository. The other half, (2.5 ml blood) was processed to extract DNA using the Genomic DNA Mini-Prep Kit (Zymo Research, California, USA) following the manufacturer’s protocol. The genomic DNA was stored at -80 °C as a component of the biorepository. The unfractionated blood and nucleic acid were aliquoted into three tubes each and stored in a separate -80 °C freezer for backup.

### Tumour specimen retrieval, processing, and storage

Routinely collected cervical cancer tumour specimens were retrieved from the respective diagnosing pathology laboratory and stored as part of this biorepository. Access to the tumour biospecimens at the diagnosing laboratory was granted by presenting the donor’s signed informed consent form to enrol into the biorepository as well as a valid approval letter from the national ethics regulatory board. Tumour biospecimens were collected as archived formalin-fixed paraffin-embedded (FFPE) blocks. Ten milligrams of FFPE blocks were sectioned off by a pathologist, ensuring that only FFPE sections with tumour tissue were included in the biorepository. In total, 5 mg was divided into two 2.5 mg tissue vials and stored at room temperature as archival tumour tissue in separate cupboards. From the remaining 5 mg of FFPE, genomic DNA was extracted following a slightly modified manufacturers’ protocol from the Zymo genomic DNA extraction FFPE kit (Zymo Research, California, USA). Paraffin was removed by adding a Xylene-based deparaffinization solution and incubating at 55^ο^C for 30 min. Purified genomic DNA was eluted in DNase/RNase Free water (pH = 7.0) and stored at -80 °C as a component of the biorepository.

### Genomic DNA quality, quantity, and integrity check

Genomic DNA quantity and purity were assessed using a Nanodrop™ Spectrophotometer 2000/2000c (Nano-drop™, ThermoFisher, Denver, USA). The Nanodrop™ calculates the concentration of DNA based on the fluorescence of a dye that binds to double-stranded DNA at 260 nm (nm) absorbance [37]. The purity of DNA was evaluated at 260/280 absorbance ratios, for protein contamination and 260/230 for guanidine contamination. DNA purity of 1.7–2.0 was acceptable for the biorepository. DNA integrity was also evaluated using gel electrophoresis.

## Biospecimen and data processing and infrastructure

### Standard operating procedures

Standard operating procedures (SOPs) for data and biospecimen collection, labelling, handling, transport, processing, and storage were developed to ensure uniform and reproducible handling procedures. Quality assurance and quality control measures were harmonised, in accordance with National Cancer Institute’s Best Practices for Biospecimen Resources. The International Society for Biological and Environmental Repositories (ISBER)’s self-assessment tool (SAT) [38] was used to determine compliance or non-compliance with the best practices for biorepositories. The SAT is an online, self-administered, risk-based algorithm, quality management system that evaluates the organisation, governance, security, equipment qualification, characterisation of biospecimens, data annotations, and SOPs for contamination control and cold chain validation. The SAT is conducted annually as per ISBER recommendations. In addition, the national ethics review committee, the Medical Research Council of Zimbabwe also performs continual reviews of the protocols and standard operating procedures of the biorepository on an annual basis. Deviations from the SOPs, as well as adverse occurrences such as failing equipment, were recorded in the incident log sheet.

### Biorepository space and storage infrastructure

The biorepository space is in an access-controlled laboratory. All the storage spaces used in the biorepository are shared spaces within the University of Zimbabwe Faculty of Medicine and Health Sciences, and so, institutional risk management strategies were adopted.

### Freezer management

The blood biospecimens and genomic DNA were stored in a lockable -80 °C freezer that is maintained and regularly monitored to maintain the temperature at -80 °C. A trained laboratory technician performs regular checks for temperature, ice build-up, gasket damage and rubber seals, battery health and the backup unit, and freezer gas levels twice a month. Extensive freezer maintenance is done on an annual basis. All freezers are connected to backup emergency power and carbon dioxide supplies that go on immediately when normal supplies are cut off. Backup units are provided by the institution and serviced regularly as part of the institutional mandate. In cases of freezer failure, all biospecimens will be moved to a backup -80 °C freezer and reports of the freezer failure will be recorded in the incident log sheet, including the highest temperature of the failing freezer and the time of biospecimen rescue.

### Data infrastructure

All paper-based de-identified donor data collection sheets as well as informed consent forms were stored in distinctive lockable filing cabinets. Clinical and phenotype data were then captured onto a commercially available and password-controlled spreadsheet (Microsoft Excel, Microsoft Corporation, Redmond, Washington, USA). An external hard drive was also used as an additional backup for the de-identified phenotype data, and the hard drive was kept in a lockable cupboard. The server on which this data is stored, as well as the protocols used, are backed up on a commercially available cloud storage platform (OneDrive, Microsoft Office, Microsoft Corporation, Redmond, Washington, USA). The cloud server is synchronised regularly per institutional regulations. Genotype data that was extracted from the analysis of the biospecimens and integrated with the demographic and phenotype data in STATA (StataCorp LLC, Station College, TX, USA) and GraphPad (Prisma, San Diego California, USA) are also available.

### Biorepository governance

Based on the nature of the cohort, local setting and type of research, a governance framework was developed following local and national ethics guidelines – Medical Research Council of Zimbabwe (MRCZ), Research Council of Zimbabwe (RCZ); and the H3 Africa Consortium Data Access and Release Policy [39].

## Results

### The cervical cancer biorepository data

Sociodemographic, risk factors, gynaecological, and family history data were collected for all 410 donors. A summary of the biorepository donor characteristics shows the mean (± SD) age to be 52 (± 12) years. Forty-five percent of the group was HIV positive, with a low history of genital warts (10%). Tumour staging distribution, as per International Federation of Gynecology and Obstetrics (FIGO) criteria was stage I (*n* = 59; 15%), IIA/B (*n* = 180; 44%), IIIA/B (*n* = 193; 47%), and IVA (*n* = 25; 6%). The most frequently detected histological type of cervical cancer was squamous cell carcinoma (83%), followed by adenocarcinoma (9%) and adenosquamous carcinoma (3%).

After the 12-month follow-up, complete data for outcomes of interest were obtained for 252 (61.5%) donors (Table 3). During the follow-up period, 158 (38.5%) had unavailable data due to loss to follow-up (LTFU). Reasons for LTFU included disease progression which led to referral for care in another facility *e.g.,* hospice (*N* = 12 out of 158); economic challenges that resulted in patients not attending treatment (*N* = 120 out of 158); as well as missing patient folders (*N* = 26 out of 158). Blood was successfully drawn from 311 (~ 76%) donors and consequently, 311 (~ 76%) genomic host DNA isolates are available. In total, 258 (63%) matched tumour biospecimens were retrieved, and corresponding genomic tumour DNA is also available in the biorepository.

**Table 3:** A catalog of the available data in the phenotype data repository per time point

TIT	Number of donor data available per time point (months)			
	**Pre-treatment**	**3**	**6**	**12**
DVT	410	400	320	252
Ototoxicity	410	375	300	252
Peripheral Neuropathy	410	375	300	252
Renal Injury	410	400	350	252
VVF	410	400	350	252
Vaginal stenosis	410	400	350	252
Vestibulopathy	410	375	300	252
Survival or mortality				252
Disease status				252

*DVT* Deep vein thrombosis, *VVF* Vesicovaginal fistula, *Disease status* Disease free progression, relapse, or disease progression

A complete overview of the components of the cervical cancer biorepository illustrates that complete data, *i.e.,* matched phenotype, host and tumour data are available for 149 (36%) donors. Matched host data and phenotype data are available for 201 (49%) donors, matched host and tumour data are available for 238 (58%) donors, as well as matched tumour data and phenotype data are available for 240 (59%) donors. These data are important to understand trends in pharmacogenetics, and genetic and non-genetic risk factors of cervical cancer in Zimbabwean women, respectively.

### Data and biospecimen access and governance

All research protocols using biospecimens and data from the biorepository need to adhere to frameworks for best practices in human research and the researchers need to be certified by the international ethical, scientific, and practical standards board, Good Clinical Practice established by the National Institute for Health and Care Research [36]. Applicants are required to adhere to the benefit-sharing terms of the biorepository, this includes citing the biorepository in any subsequent publications, intellectual property rights, establishing research partnerships, capacity building of the biorepository *i.e.* donations in the form of reagents, equipment, and training for personnel.

**Fig. 2:**
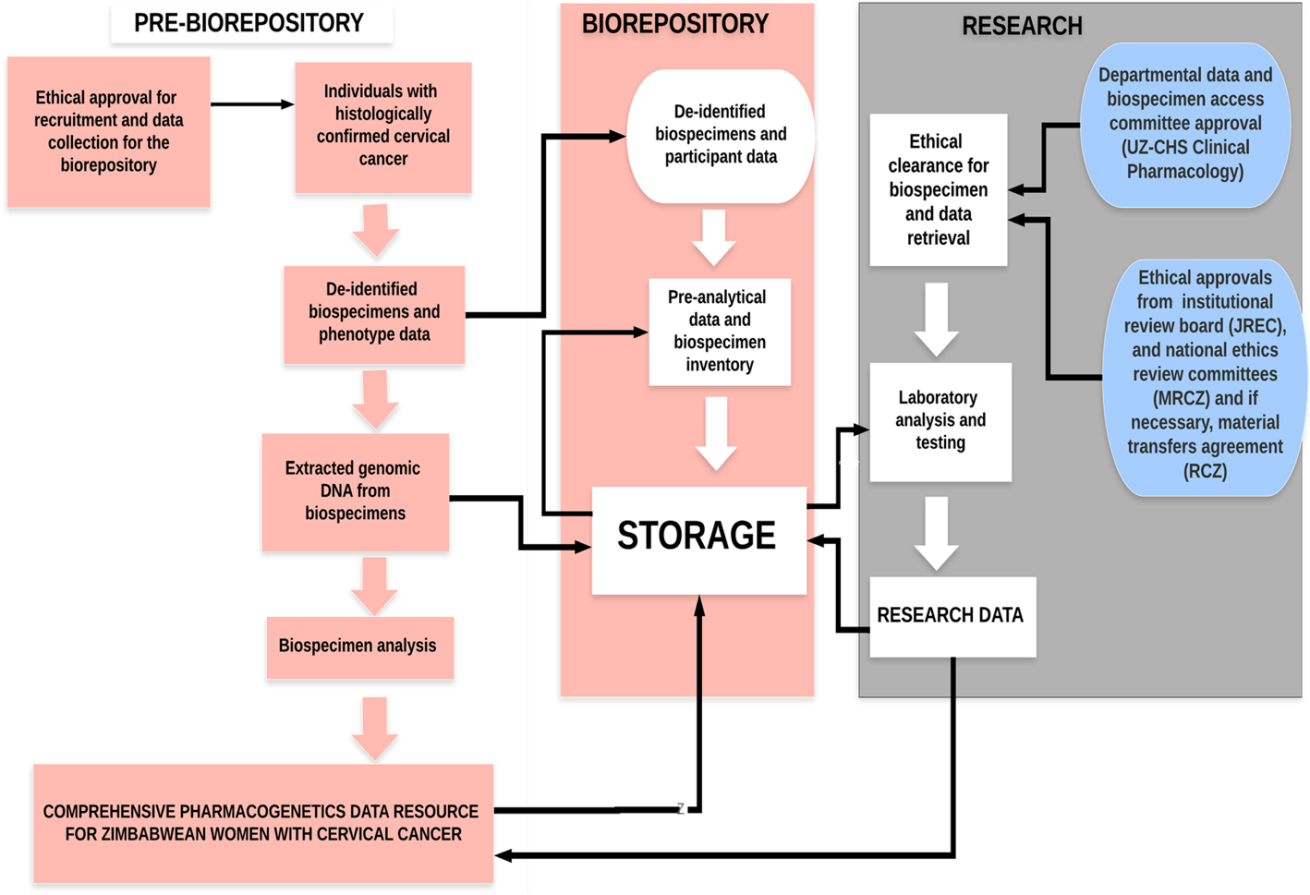
Proposed framework and governance of the cervical cancer biorepository

To safeguard the biorepository data, harmonise governance and decide on access and use of the data and biospecimens in the biorepository, major stakeholders at departmental, institutional, and national regulatory custodians will be engaged to establish a biospecimen and data access committee. The biospecimen and data access committee will be comprised of the biorepository steering committee members, as well as institutional and national ethics review committee members. Applications to use biospecimens and data in the biorepository will first go through the biospecimen and data access committee, then to the institutional ethics review board, JREC (where the biorepository is housed), and finally to the national ethics review committee, MRCZ (Fig. 2). Researchers intending to access data/biospecimens from other countries will also be required to have a valid Materials Transfer Agreement (MTA) from the Research Council of Zimbabwe (RCZ), as per the data sharing laws for human research data and biospecimens in Zimbabwe. An MTA is a bilateral agreement between the biorepository and the researcher, laying out rights, obligations, and intellectual property agreements, while also ensuring the researcher will respect donor consent and confidentiality. These synchronised and multi-tier efforts are to ensure ethical conduct in future research and mutual benefits between the communities under investigation and the future researchers. Following approval, researchers will be granted access only to phenotype data and biospecimens relevant to their specific research objectives. Applications can be amended to include or exclude other aspects of the research, subject to approval from the biospecimen and data access committee as well as the national ethics review boards. Rejected requests can be appealed to the biospecimen and data access committee as well as the national ethics review boards.

### SWOT analysis of the biorepository

**Fig. 3:**
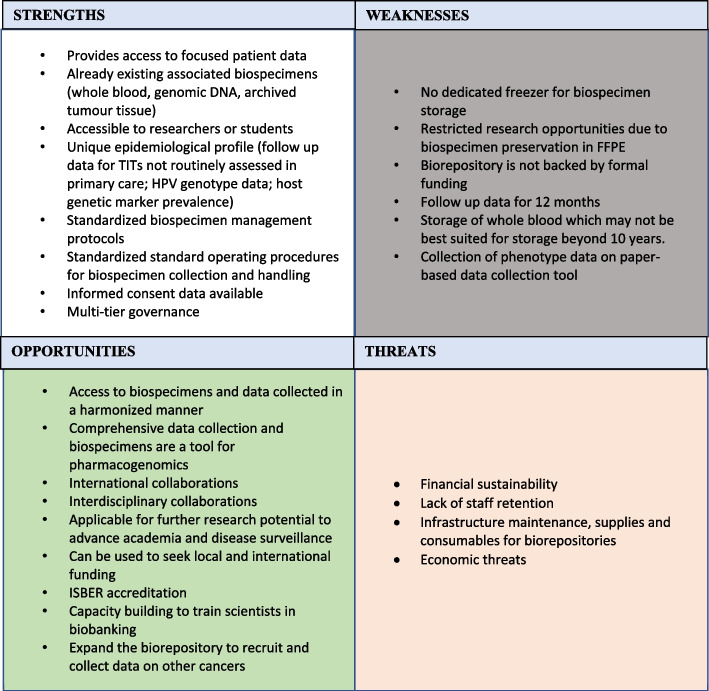
The SWOT analysis of the biorepository

Strengths, weaknesses, opportunities, and threats (SWOTs) of the biorepository were also analysed on an annual basis for rectifiable improvements and risk minimization (Fig. 3). This biorepository provides readily accessible biospecimens, as well as novel phenotype data *e.g.,* peripheral neuropathy and ototoxicity in cancer in Zimbabwe. Consequently, it has great potential for local and international collaborations as well as funding opportunities to develop this repository into a self-sustainable resource with ISBER accreditation. However, follow-up data for this biorepository is until 12 months, which may limit future studies. Lack of formal funding and economic sustainability as well as lack of staff retention due to poor remuneration in Zimbabwe are significant threats to the progress of the biorepository.

### Contribution of the biorepository to date

The goal of this cervical cancer biorepository was to establish a resource with impacts that are far-reaching to improve care for cervical cancer in Zimbabwe and can be used to generate data that further confirms the diversity of Africans. To this point, data obtained from this biorepository has contributed to several studies [12, 40]. Specifically, the phenotype data has been used to analyse the pain management strategies for cervical cancer in Zimbabwe [12] and has the potential to reshape cancer pain management in the country. DNA from the tumour biospecimens was used to determine human papillomavirus (HPV) genotypes and to compare high-risk human papillomavirus (HPV) genotypes in women of differing HIV status [40]. This work illustrated the HPV pathogenic diversity in Zimbabweans compared to other populations. For example, the HPV35 genotype was found in high prevalence (26%) in this cohort, compared to previously published data from Caucasian (2%) and Asian populations (< 1%) [40]. Other ongoing unpublished work includes a pharmacogenomics study associating host genetic variations with response to treatment, relapse and survival using the phenotypes in Table 3.

## Discussion

Cancer incidence and mortality in Zimbabwe continue to rise, expanding the need to establish vital research and surveillance tools for clinical translation and improved patient care. Biorepositories, a critical resource to systematically collect well-annotated data and biospecimens are fundamental to bridging this gap. This study described the establishment of the first formal and comprehensive cervical cancer biorepository comprising matched biospecimens and donor phenotype data collected using harmonised technical methods for Zimbabwean populations. This initial establishment of the biorepository with processes set-up from patient recruitment to genomic analysis serves as a tool for future work with the goal of studying reproductive cancers in Zimbabwe.

The current study employed broad consent in recruitment, resonating with other studies from SSA [29]. Broad consent permits researchers to re-use biospecimens and data without the need to re-contact donors, within the confines of ethical and legal regulations stipulated by the governing authority [41]. Consent models of best fit for undefined storage periods are long debated, and proponents for this model describe broad consent to be the most feasible way to recruit donors into biorepositories in resource-limited settings [42, 43]. This is because donor recontacting requires finances, logistics, time, and other resources, that may already be limited in developing settings and can even delay research. In addition to financial and logistical limitations, seeking study-specific consent from donors may also affect attrition rates in the repository, and limit available data to adequately answer research questions. However, the detractors of broad consent argue that this model impedes donor trust and undermines the core principle of informed consent- to inform the donor of all the uses for their respective data/ biospecimens [43–45]. Tindana and collaborators illustrated that broad consent would be of great benefit to avoid these aforementioned factors raised against broad consent, by promoting the trustworthiness of the research institutions, researchers, protocols in performing the research, effective communication with the community at large and ensuring the community benefits from the outputs of the work [46]. Furthermore, studies that have interrogated the donor’s perspective on broad consent have also provided contraindicative conclusions. In most studies, donors view broad consent as an acceptable mode of consent provided the intention to store and share data/biospecimens is clearly stated from recruitment [47, 48]. Such research is currently underway in Zimbabwe under the H3Africa initiative and would be important to understand the perspective of Zimbabwean donors in consent models in this context [20].

In this cohort, TITs followed up routinely in cervical cancer care include DVT, renal injury, haematological complications, VVF, radiation proctitis and vaginal stenosis. Consequently, retrospective data on the occurrence of these TITs in Zimbabwean women with cervical cancer is available [49–52]. This current biorepository provides access to prospective data for these routinely monitored TITs. In addition, TITs that are yet to be reported in Zimbabwean cancer patients—ototoxicity, peripheral neuropathy and vestibulopathy are also described in this biorepository. These TITs have debilitating effects that can range from physical disability or in extreme cases, death [53, 54]. Therefore, this biorepository uniquely offers novel data that can potentially be used to identify neglected TITs that should be monitored at point of care. Similarly, limited TITs are followed up routinely in other African countries, and little data on peripheral neuropathy and ototoxicity in other Africans have also been previously reported [55]. Therefore, this biorepository offers phenotypic data that can be used to estimate the burden of these TITs in countries of similar socioeconomic status.

Moreover, this biorepository also provides matched phenotype and host specimen data that can be used to identify host genetic polymorphisms and somatic variations towards a robust framework for pharmacogenomics of cervical cancer study. As it stands, there are no established pharmacogenetic markers for cervical cancer, and yet these data are important to develop novel targeted therapies for the management of cervical cancer.

This biorepository was established without the backing of formal funding, although this is identified as a weakness in the SWOT analyses, this work describes the establishment of a biorepository on a limited budget and resources. The vital components of this biorepository focused on the determination of germline and somatic variants and their impact in pharmacological response in cancer diagnosis, prognosis, and management. Consequently, this biorepository provides a set of processes in data study design, biospecimen and data methods and processes which can be tailored for a cancer pharmacogenomics study whereby various sample types and specific clinical information is critical for a comprehensive dataset. The approach described can also be adopted in other resource-limited settings which can start off as small-scale biorepositories developed in accordance with national and international best practices standards, with the potential to grow into large-scale biorepositories.

In particular, this biorepository provides an opportunity to foster local and international collaborative relationships, aspects that are both desirable and crucial to biomedical advancement and to building sustainable biorepositories in Africa. A study conducted in Zimbabwe found that there is a lack of coordinated use of existing infrastructure and resources and poor resources for research education, thus most researchers tend to collect fresh biospecimens and data, which tends to narrow the perspective and expertise of the research [21]. Collaborations have been shown to promote resource efficiency, improve the quality of research, easily attain statistical power, while also maintaining the integrity of the ethical and legal frameworks that the present biorepository are built on. In future, we aim to develop a publicly available website with detailed information on the biorepository catalogues, activities, governance, and services; as well as to develop a database for smaller collections of biospecimens and associated phenotype data. This is an important step to increase the visibility of biorepositories and create a bridge for collaborative networking.

## Challenges and recommendations

Data presented here highlights a predicament faced by many in low-resource settings, such as LTFU. Approximately 40% of donors were LTFU within 12 months, which translated to incomplete data. The main reason for the LTFU was related to financial constraints due to the economic burden of seeking medical care in Zimbabwe [56, 57]. Therefore, biorepositories of this calibre offer an alternative resource for new studies which may need similar types of biospecimens, and ready-made SOPs for biospecimen and data collection.

Clinical and phenotype data were collected on paper data collection sheets, which can be time-consuming [58–60]. Although in this biorepository, quality assurance and quality control were conducted by multiple independent individuals, paper-based data collection has lower accuracy (93%) when compared to electronic data collection (99%) [61, 62]. Automated systems including electronic data collection reduce the risk of errors, duplication, and save time for processes such as data cleaning [63–66]. Electronic reports are favoured by donors and could also be a motivator for enrolment [67].

Shared resources *e.g*., laboratory freezer and lockable cabinets are used in this biorepository. This storage model is sufficient at present when the collection of data is relatively small, however, there is a need to expand on space as the biorepository expands. Particularly, procuring dedicated freezers for this biorepository will be key to ensuring systematic organisation and easy tracking of the biospecimens. Acquisition of formal funding is key for economical sustainability as well as to ensure swift logistical operations of the resource and the outputs of such a biorepository are a crucial foundation for obtaining economic support [68–70]. Therefore, it is hoped that ongoing projects utilising this dataset will reiterate the importance of this resource in informing cervical cancer care and motivate for the access of funds to sustain and expand the biorepository.

## Sustainability goals

In the establishment of the cervical cancer biorepository, there was a non-profit clause that was included in the informed consent process, and as a result, this repository does not operate on a fee-for-service model as recommended by ISBER best practices. Instead, this biorepository relies on establishing mutually beneficial partnerships with end-users where users of the biorepository will be required to donate toward the sustainability of the bioresource. Donations can be in the form of reagents, laboratory equipment, technological inputs, and capacity building for the biorepository personnel. As the biorepository continues to grow and by consulting with institutions and regulatory authorities, a fee-for-service may be implemented in a transparent manner, to enable continuity of high-quality biospecimen and data collection, processing, storage, and quality control measures.

## Limitations of the biorepository and future perspectives

1. The data was collected at a single site. In future, this resource will also collect data from multiple sites to ensure adequate representation of the socio-demographic diversity in Zimbabwe.
2. This study stored whole blood as unfractionated biospecimens, which may limit the amount of DNA that can be extracted, 10 years after the sample was collected. In future, backup buffy coats will be prepared and stored long-term for future analyses. Furthermore, in future, the genomic DNA extracted will be eluted in an elution buffer to increase durability of the DNA.
3. The current data consists of cervical cancer cases only. In future, matched healthy controls will also be recruited for case–control studies.
4. The current study stored the data on a Microsoft Excel spreadsheet. As the cohort continues to grow, the data will be exported onto a database such as REDCAP^73^, an affordable, secure, web-based data management tool. The authors are also exploring suitable laboratory information management systems which can integrate meta-data with genomic data generation.

## Conclusion

Cancer genomics and pharmacogenomics provide leading examples for the application of personalised medicine. As more discoveries of somatic genomic variation are underway, such a repository offers access to biospecimens for future research. This work demonstrates a potential framework for the establishment and implementation of a biorepository for genomics and pharmacogenomics of cancer on a limited budget in a resource-limited environment.

## Supplementary Information

**Additional file 1:** **Supplmentary Table 1.** Comparison of the with ISBER best practice with the cervical cancer biorepository practice.

## Data Availability

The datasets generated and/or analysed during the current study are not currently available and will be made available upon reasonable request due to assurances made to the donors during the informed consent process. All donor data and biospecimens were informed that the data and biospecimens would only be made available to researchers with contact from the corresponding author on reasonable request.
