# Supplementary material for: A cervical cancer biorepository for pharmacogenomics research in Zimbabwe
Source: BMC Cancer. 2022 Dec 16;22:1320. doi: 10.1186/s12885-022-10413-w (PMC9756582; doi:10.1186/s12885-022-10413-w)
Supplement: Supplementary file 1 — Additional file 1: Supplmentary Table 1. Comparison of the with ISBER best practice with the cervical cancer biorepository practice. [file 12885_2022_10413_MOESM1_ESM.docx]

**Supplmentary Table 1: Comparison of the with ISBER best practice with the cervical cancer biorepository practice**

| **ISBER Best Practice** | **Cervical cancer biorepository practice** |
| --- | --- |
| Governing body | **✓** |
| Written policies covering full repository life cycle | **✓** |
| Readily available specimen and data governance | **✓** |
| Inclusion and exclusion criteria are clearly outlined | **✓** |
| Quality management systems including feedback from researchers | **✓** |
| Clear guidelines of services provided, operational times, after hour emergencies | **✓** |
| Clear organizational structure and job description | **✓** |
| Disaster recovery plans if the repository is in extreme environmental conditions *e.g.,* tsunami/earthquake prone areas |  |
| Temperature control for refrigerators/ biorepository spaces equipped with appropriate alarms for monitoring, appropriate lighting, back-up power and back up cooling systems | **✓** |
| Twenty-four hour monitored security systems | **✓** |
| Visitors log sheet |  |
| Fire and hazard prevention plan, adequate supplies of extinguishers and an emergency contact staff | **✓** |
| Repository inventory at the site of the biorepository | **✓** |
| Duplication of specimen location in different location | **🗶** |
| Relocation strategy in case of emergency, including map to locate biorepository items | **✓** |
| Appropriate personnel protective equipment for -80 degrees Celsius access | **✓** |
| Barcodes for specimen tracking | **🗶** |
| Storage of FFPEs at room temperature | **✓** |
| Review SOPs annually or when policy/methods change, and train staff accordingly | **✓** |
| Use a metric system with key performance indicators for quality assurance | **🗶** |
| Good documentation practices as per an appropriate quality management system | **✓** |
| Offsite record back up system | **✓** |
| Adequate training for personnel, with applicable Good Practices certification | **✓** |
| Cost recovery policies | **🗶** |
| Traceable validation systems | **✓** |
| Periodic database audit to ensure data accuracy | **✓** |
| Frequent backup of biorepository data onto a “cloud” | **✓** |
| Labelling of biospecimens with an ID that reflects storage location in the repository | **🗶** |
| Cold chain management | **✓** |
| Documented pre-analytical procedures including data collection, process, shipping, and storage, to be shared with end-users | **✓** |
| Documented freeze/thaw cycles for biospecimens in the repository | **✓** |
| Data and biospecimens collection should not interfere with patient care | **✓** |
| PPE should be worn during biospecimen collection, handling, transport and processing | **✓** |
| Blood specimens should be processed and stored in 24hours of draw | **✓** |
| A second independent quality control check should be performed at specimen retrieval | **✓** |
| Informed consent which also describes return of research results should be described | **✓** |
| Collection, storage, distribution, use and disposal of specimens in the repository should respect perspectives and traditions of donors | **✓** |
| Repositories should follow national/federal, regional, local and international guidelines related to access and benefit sharing | **✓** |
| Written policies governing access, use and transfer of specimens of data | **✓** |
| Established data sharing policies including intellectual property, informed consent, ethical and privacy standards | **✓** |
| Clearly defined criteria for evaluating requests for access to the biorepository | **✓** |
| Benefit sharing agreements to establish acknowledgement in publications | **✓** |
| Documented disposition of specimens or collections through destruction of transfer to a new custodian, along with related documents in documents archives | **✓** |

Footnote:: ✓ yes; 🗶 no
